# Spectroscopic and Thermodynamic Elucidation of COD Adsorption Mechanisms on a Porous Carbon-Based Resin

**DOI:** 10.3390/molecules31081319

**Published:** 2026-04-17

**Authors:** Yali Wang, Chenghu Wang, Liqing Fan, Miao Li, Ruilin Feng, Yanke Chen

**Affiliations:** 1 School of Chemistry and Chemical Engineering, Yulin University, Yulin 719000, China; 2 Shaanxi Shuangyi Coal Chemical Technology Industrial Group Co., Ltd., Jinyuan South Road, Shenmu High-Tech Industrial Development Zone, Yulin 719319, China

**Keywords:** semi-coking wastewater, COD, porous carbon resin, adsorption mechanism, π–π interactions, hydrogen bonding, adsorption kinetics, industrial wastewater treatment

## Abstract

Semi-coking wastewater generated during coal pyrolysis contains extremely high concentrations of refractory organic pollutants, resulting in elevated chemical oxygen demand (COD) and posing significant environmental risks, making efficient COD removal a critical challenge for sustainable wastewater treatment in the coal chemical industry. In this study, a porous carbon-based resin (XDA-1G) was investigated as an adsorbent for COD removal from semi-coking wastewater. The adsorption performance and underlying mechanisms were systematically evaluated through adsorption isotherm, kinetic, and thermodynamic analyses, combined with structural characterization using FTIR, XPS, BET, XRD, and SEM–EDS. The resin exhibited a high COD removal efficiency of up to 91% with a maximum adsorption capacity of 2182 mg g^−1^. Kinetic analysis followed the pseudo-second-order model, while the Freundlich isotherm best described the equilibrium behavior, indicating heterogeneous adsorption. Thermodynamic parameters confirmed that the adsorption process is spontaneous and endothermic. Spectroscopic and structural analyses revealed that COD removal is mainly governed by synergistic mechanisms including π–π interactions between aromatic pollutants and the carbon framework, hydrogen bonding with oxygen-containing functional groups, and pore filling within the hierarchical porous structure. These findings demonstrate the strong potential of porous carbon-based resins as efficient adsorbents for treating high-strength industrial wastewater.

## 1. Introduction

Effluents produced during low- to medium-temperature coal pyrolysis represent a major source of environmental pollution due to their chemically intricate composition and extremely high concentrations of refractory organic compounds [1]. Among these contaminants, the COD is particularly elevated—often exceeding 50,000 mg L^−1^—indicating the presence of stable, toxic organic species that resist biodegradation [2]. The elevated COD levels primarily originate from phenolic substances, oxygenated aromatics, and other persistent organics, which imposes a substantial oxygen demand upon discharge into aquatic environments, leading to oxygen depletion and serious ecological consequences [3,4,5].

Reducing COD is therefore a critical goal in the treatment of semi-coking and other coal-chemical wastewaters. Among various technologies—including advanced oxidation [6,7], biological treatment [8,9], and membrane separation [10]—adsorption has emerged as a particularly attractive approach due to its operational simplicity, efficiency, and adaptability to high-strength industrial effluents [11,12]. Unlike biological systems, adsorption does not require microbial acclimation or chemical oxidants, and can efficiently remove a wide spectrum of organics under diverse environmental conditions.

The effectiveness of adsorption is strongly governed by the physicochemical properties of the adsorbent. Carbon-based resins, in particular, have demonstrated remarkable potential for the removal of organic contaminants owing to their large specific surface area, adjustable pore architecture, and abundance of functional groups capable of diverse interactions [13]. Previous studies have shown that phenolic resins can effectively adsorb various micropollutants, such as paracetamol, primarily through π–π interactions and hydrogen bonding mechanisms [14]. Likewise, ion exchange [15,16] and functionalized polymeric resins [17] have been successfully employed to eliminate organic matter and other pollutants from complex aqueous environments, highlighting their versatility and strong affinity for both polar and nonpolar organic compounds.

Recent innovations in resin design, particularly those involving surface modification [18,19,20], have markedly enhanced adsorption performance and selectivity toward organic pollutants. The incorporation of porous nanomaterials into resin matrices not only increases specific surface area and pore accessibility but also introduces additional active sites for adsorption [21,22]. Similarly, chemical modification with oxygen- and nitrogen-containing functional groups enriches the surface with polar sites capable of hydrogen bonding, electrostatic attraction, and π–π interactions, thereby improving the affinity toward aromatic and oxygenated organic compounds [19,20,23]. These functionalized resins exhibit enhanced adsorption kinetics and stability, even under harsh wastewater conditions. Furthermore, the integration of resin-based adsorption with biological processes—such as anaerobic digestion—has emerged as a promising hybrid strategy that combines physicochemical adsorption with biodegradation [24]. This coupling not only broadens the range of removable pollutants but also promotes energy recovery through the conversion of adsorbed organics into biogas, offering a sustainable pathway for the treatment and valorization of high-strength industrial wastewaters.

Increasing evidence emphasizes that the fine-scale pore architecture of resins plays a decisive role in determining their adsorption efficiency toward phenolic and aromatic contaminants. Polymeric adsorbents possess extensive BET surface areas and well-developed microporous networks and have been shown to achieve markedly higher phenol uptake compared with materials dominated by meso- or macropores [25,26]. The abundance of micropores not only increases accessible adsorption sites but also strengthens van der Waals and π–π interactions with hydrophobic organics, thereby enhancing selectivity and capacity. The XDA-1G resin, a representative non-polar adsorbent featuring a primarily microporous framework with limited macropore contribution, exhibits exceptional affinity for aromatic and oxygenated organic molecules. Its adsorption behavior arises from multiple cooperative mechanisms, including hydrogen bonding between carbonyl or hydroxyl moieties on the resin surface and functional groups of COD species, π–π stacking among aromatic rings, and hydrophobic interactions within the carbonaceous matrix. These synergistic effects endow XDA-1G with superior structural stability and adsorption persistence across a broad range of temperatures and pollutant concentrations [27]. Compared with conventional granular activated carbon, such resins provide improved molecular selectivity, chemical resilience, and reusability, making them efficient and economically attractive candidates for large-scale COD abatement in complex industrial effluents.

In this study, a commercially available carbon-based resin with hierarchical porosity and abundant surface functionalities was employed to remove COD from semi-coking wastewater. Unlike most studies that focus on dilute phenolic solutions, this work targets real high-strength wastewater systems with COD exceeding 50,000 mg L^−1^. The study aims to: (1) characterize the resin’s structural and chemical evolution before and after adsorption using FTIR, XPS, XRD, BET, and SEM–EDS; (2) systematically evaluate the adsorption behavior of the resin under different operational conditions; and (3) elucidate the adsorption mechanism through comprehensive modeling of isotherms, kinetics, and thermodynamics. The findings provide molecular-level insight into the interactions between the resin and complex organic pollutants, offering a basis for the development of high-capacity, regenerable adsorbents for industrial wastewater purification.

## 2. Results and Discussion

### 2.1. Characterization of the Materials

#### 2.1.1. FTIR

**Figure 1:**
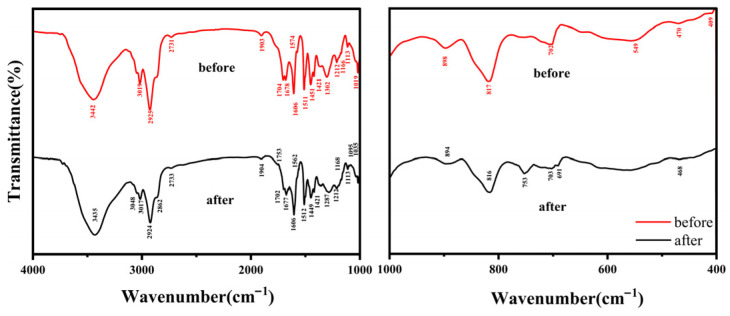
FTIR spectra of the resin before and after adsorption of semi-coking wastewater.

Figure 1 compares the FTIR spectra of the pristine resin (“before”) and the resin after adsorption of semi-coking wastewater (“after”). The observed absorption bands and their assignments are presented in Figure 1. After adsorption, slight shifts in band positions are observed, indicating structural and interfacial changes in the resin associated with the interaction of oxygen-containing organic species.

A broad absorption band centered at 3442 → 3435 cm^−1^ (O–H stretching) slightly shifts to lower wavenumber after adsorption, reflecting enhanced hydrogen bonding between resin polar groups and adsorbed phenolic and carboxylic species [28,29]. The weak high-frequency band at 3750 → 3729 cm^−1^ corresponds to isolated hydroxyls [30], whose decreased intensity indicates their involvement in surface hydrogen bonding.

The aromatic C–H stretching band near 3049 → 3048 cm^−1^ becomes more pronounced [31], while the aliphatic C–H bands at 2925 → 2924 cm^−1^ and 2863 → 2862 cm^−1^ also increase, indicating the adsorption of both aromatic and aliphatic organic compounds from the wastewater [32]. The emergence of a distinct carbonyl band at 1730 cm^−1^ and a shoulder near 1753 cm^−1^ after adsorption—absent or weak before treatment—confirm the uptake of oxygenated organics such as esters, acids, and aryl-ketones [33].

Absorption bands at 1511 → 1512 cm^−1^ and 1451 → 1449 cm^−1^ correspond to aromatic skeletal vibrations and C–H bending, suggesting the accumulation of benzene-type structures. The absorption bands at 1302 → 1287 cm^−1^ and 1212 cm^−1^ are attributed to phenolic C–O and C–O–C stretching, respectively, indicating enrichment of oxygen-containing aromatic molecules. In the fingerprint region, bands at 1113 cm^−1^, 1019 → 1035 cm^−1^, and 898 → 894 cm^−1^ are assigned to C–O stretching and aromatic C–H bending, while those at 817 → 816 cm^−1^ and 702 → 703 cm^−1^ correspond to out-of-plane C–H deformations in substituted aromatics. Weak lattice modes at 470–468 cm^−1^ and 422 cm^−1^ remain, consistent with the resin backbone vibrations.

The observed red-shift of hydroxyl absorption bands, the emergence of carbonyl bands, and changes in aromatic-related vibrations suggest that COD removal is primarily governed by physical interactions, including (i) hydrogen bonding between phenolic and carboxylate groups and functional moieties on the resin surface, and (ii) π–π interactions and dispersion forces between aromatic structures in the wastewater and the carbon-rich resin matrix. These spectral features indicate that the resin effectively adsorbs phenolic, aromatic, and oxygenated species—the dominant contributors to COD in semi-coking wastewater—mainly through physisorption processes.

#### 2.1.2. XPS

**Figure 2:**
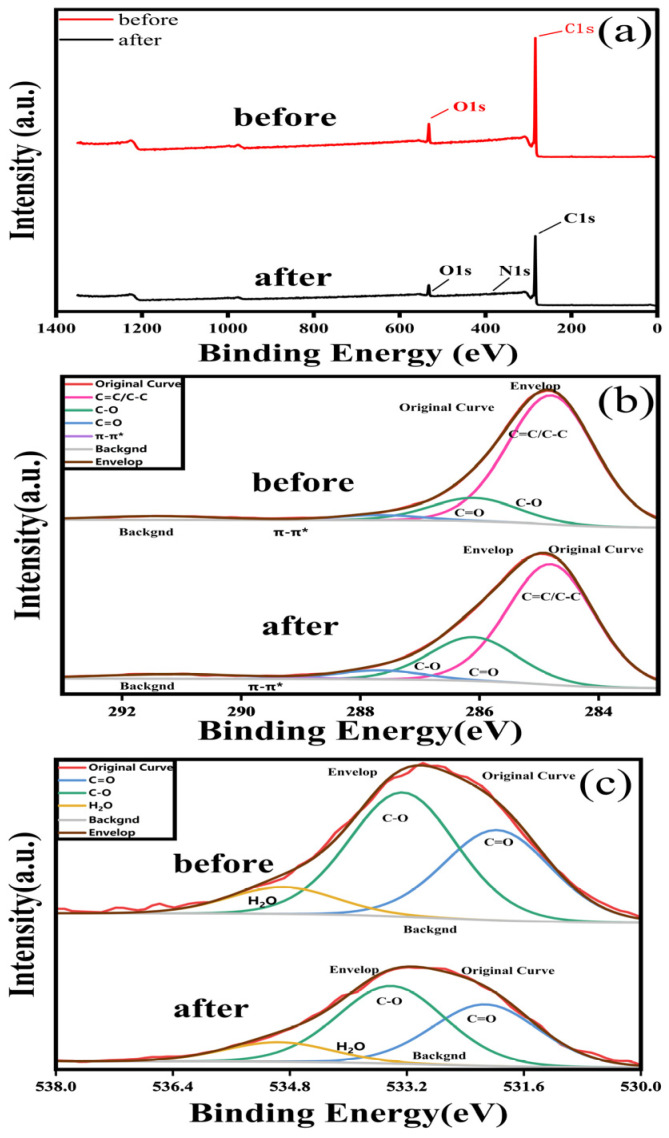
XPS spectra of the resin before and after adsorption of semi-coking wastewater: (**a**) survey spectra, (**b**) deconvoluted C 1s spectra, and (**c**) deconvoluted O 1s spectra.

The XPS survey spectra (Figure 2a) confirm that the resin surface is dominated by C and O, with trace heteroatoms. After contact with semi-coking wastewater, the overall surface atomic ratios change slightly (C1s: 92.29 → 90.63 at%; O1s: 7.71 → 7.76 at%) and a small N1s signal (1.61 at%) appears, indicating adsorption of oxygen-containing species along with nitrogen-bearing organics together (Table 1).

High-resolution C1s spectra (Figure 2b) were deconvoluted into C=C/C–C (284.8 eV), C–O (286.1 eV), C=O (287.7 eV), and a π–π* shake-up feature (~291 eV). After adsorption, the fraction of graphitic/aliphatic carbon decreases markedly (81.92 → 69.50%), whereas oxygenated carbons increase: C–O rises from 14.80 to 25.31% and C=O from 3.28 to 5.19%. The persistence of the π–π* satellite (291.4 → 291.1 eV) evidences aromatic rings on the surface and is consistent with uptake of phenolics and other aromatic oxygenates typical of semi-coking effluents.

The O1s region (Figure 2c) was fitted with C=O/O–C=O (~532 eV), C–O (~533 eV), and H_2_O/adsorbed water (535.1 eV). Following adsorption, the relative contribution of carbonyl oxygen increases slightly (37.39 → 38.71%), the C–O component decreases modestly (51.48 → 48.94%), and the high-binding water/strongly H-bonded oxygen increases (11.13 → 12.35%). All O1s components shift by ~0.2–0.3 eV to higher binding energy (e.g., C=O: 531.97 → 532.27 eV; C–O: 533.26 → 533.55 eV), indicating a more electron-deficient environment due to hydrogen bonding and/or specific interactions between the resin and adsorbates.

Collectively, (i) enrichment of C–O/C=O moieties in C1s, (ii) slight O1s upshifts, and (iii) the maintained π–π* satellite demonstrate that the resin captures oxygen-containing aromatics from semi-coking wastewater. These data support a multimodal adsorption mechanism involving π–π interactions with aromatic backbones and hydrogen-bonding/ion–dipole interactions with phenolic, carbonyl, and amine/amide functionalities that contribute to COD.

**Figure 3:**
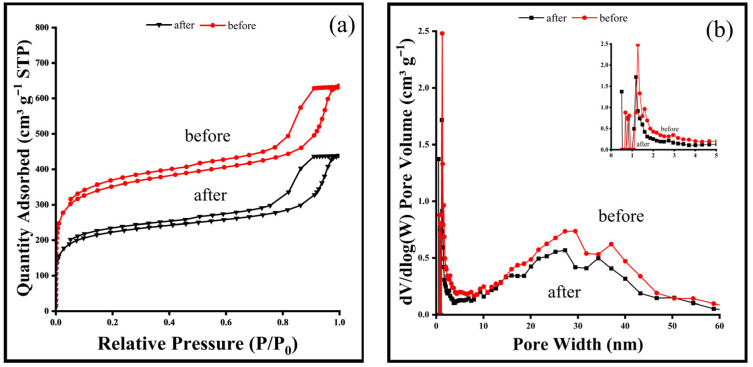
(**a**) N_2_ adsorption–desorption isotherms and (**b**) pore size distribution of the resin before and after adsorption of semi-coking wastewater.

The nitrogen adsorption–desorption isotherms of the resin before and after adsorption of semi-coking wastewater are shown in Figure 3a, and the corresponding pore-size distribution curves are presented in Figure 3b. Both samples exhibit type IV isotherms with H4-type hysteresis loops according to the IUPAC classification, indicating the presence of a hierarchical micro–mesoporous structure with slit-shaped pores [37,38]. The steep adsorption at low relative pressures (P/P_0_ < 0.1) corresponds to micropore filling, while the hysteresis at higher P/P_0_ values reflects capillary condensation in mesopores [39]. The coexistence of micropores and mesopores facilitates molecular diffusion, which is particularly beneficial for capturing a wide range of organic pollutants with different molecular sizes.

The pore-size distribution curves (Figure 3b) indicate the coexistence of both micropores (~2 nm) and mesopores (5–50 nm), suggesting a hierarchical porous structure. After adsorption of semi-coking wastewater, both surface areas and pore volumes in both the micro- and mesopore regions decrease notably, confirming that organic molecules from the wastewater occupy and partially block the pore channels of the resin. The detailed textural parameters derived from BET, BJH, t-plot, and Langmuir analyses are summarized in Table 2. The observed differences among these values arise from the use of different calculation methods, each based on distinct theoretical assumptions and applicable to different pore size ranges. Specifically, the BET method estimates surface area based on multilayer adsorption over a broad pore size range, while the Langmuir model assumes monolayer adsorption on an ideal homogeneous surface, often resulting in higher calculated surface areas. In contrast, the BJH method is primarily used to evaluate mesopore size distribution and surface area based on capillary condensation, whereas the t-plot method is more suitable for distinguishing micropore volume and external surface area. Due to these methodological differences and their sensitivity to different pore regimes, variations in the calculated surface areas and pore parameters are expected. The specific surface area, micropore area, and total pore volume of the resin show substantial reductions after adsorption. Specifically, the BET surface area decreases from 1306 to 826.0 m^2^ g^−1^, Langmuir surface area from 2339.91 to 1593.66 m^2^ g^−1^, and the BJH adsorption surface area from 324.20 to 213.02 m^2^ g^−1^ and the t-plot micropore area from 617.4 to 371.3.

Similarly, the BJH adsorption volume and the micropore volume decline from 0.5621 to 0.4211 cm^3^ g^−1^ and from 0.2505 to 0.1509 cm^3^ g^−1^, respectively. These reductions indicate that pore filling and blockage occur as organic pollutants diffuse into the resin’s micro–mesoporous network. The relatively larger decrease in mesopore volume suggests that macromolecular or aromatic compounds preferentially occupy mesopores, while smaller oxygenated molecules enter the microporous regions. The overall decrease in surface area and pore volume confirms that adsorption is not limited to surface sites but extends deep into the pore structure. Therefore, the resin’s hierarchical pore system plays a critical role in COD removal from semi-coking wastewater. Micropores provide abundant adsorption sites and strong capillary forces for small oxygenated organics. Mesopores offer transport channels that enhance diffusion and accessibility of larger aromatic molecules.

After adsorption, the occupation of these pores reflects the strong binding and accumulation of organic chemicals within the resin matrix. Combined with the FTIR and XPS analyses, these structural changes further support a multimodal adsorption mechanism involving π–π stacking, hydrogen bonding, and pore confinement effects.

#### 2.1.3. XRD

**Figure 4:**
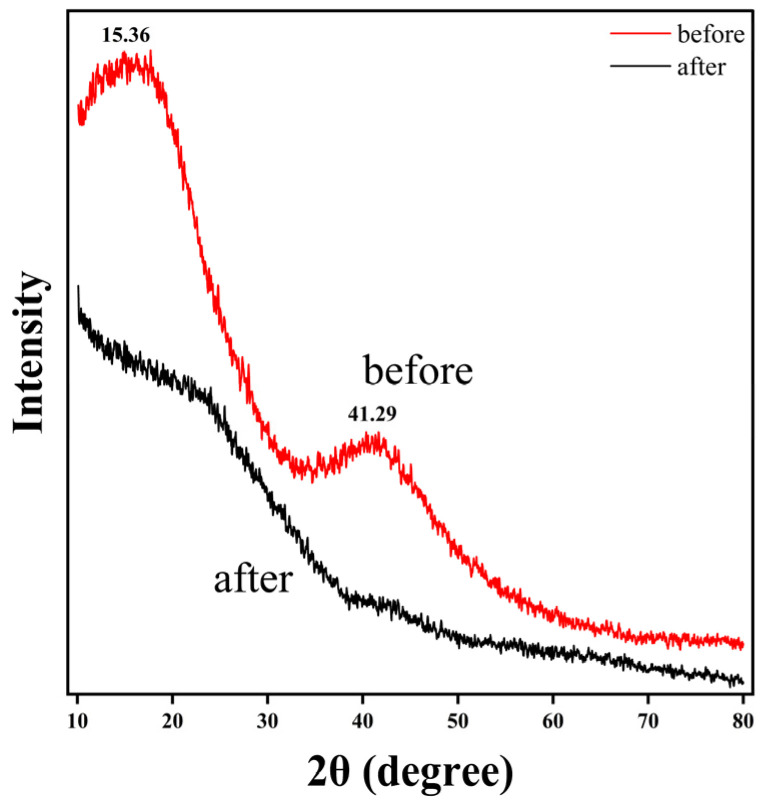
XRD spectra of the resin before and after adsorption of semi-coking wastewater.

Figure 4 presents the XRD patterns of the pristine resin (“before”) and the resin after adsorption of semi-coking wastewater (“after”). Both samples exhibit two broad diffraction peaks centered at approximately 2θ = 15.36° and 41.29°, which correspond to the (002) and (100) reflections of disordered carbonaceous structures [40]. The absence of sharp crystalline absorption bands indicates that the resin possesses an amorphous, turbostratic carbon framework, consisting of randomly stacked aromatic layers.

After adsorption, the overall diffraction intensity decreases noticeably, and the broad features become weaker and slightly shifted, suggesting increased structural disorder. This attenuation is attributed to the deposition of organic molecules from semi-coking wastewater onto the resin surface and within its pore network, which disrupts the regular stacking of carbon layers. Such behavior is typical for carbon-based adsorbents following surface functionalization or organic adsorption, reflecting enhanced heterogeneity and interlayer distortion.

The persistent broad halo near 15–20° confirms that the carbon backbone remains amorphous, while the diminished intensity of the ~41° peak implies partial coverage or interaction between π-conjugated regions of the resin and aromatic compounds in the wastewater. This interaction supports π–π stacking between the resin’s carbon layers and aromatic pollutants, consistent with the FTIR and kinetic analyses.

The XRD results verify that both pristine and spent resins are amorphous carbon materials, and that adsorption of COD species from semi-coking wastewater leads to increased disorder and surface modification without altering the fundamental carbon framework.

#### 2.1.4. SEM

**Figure 5:**
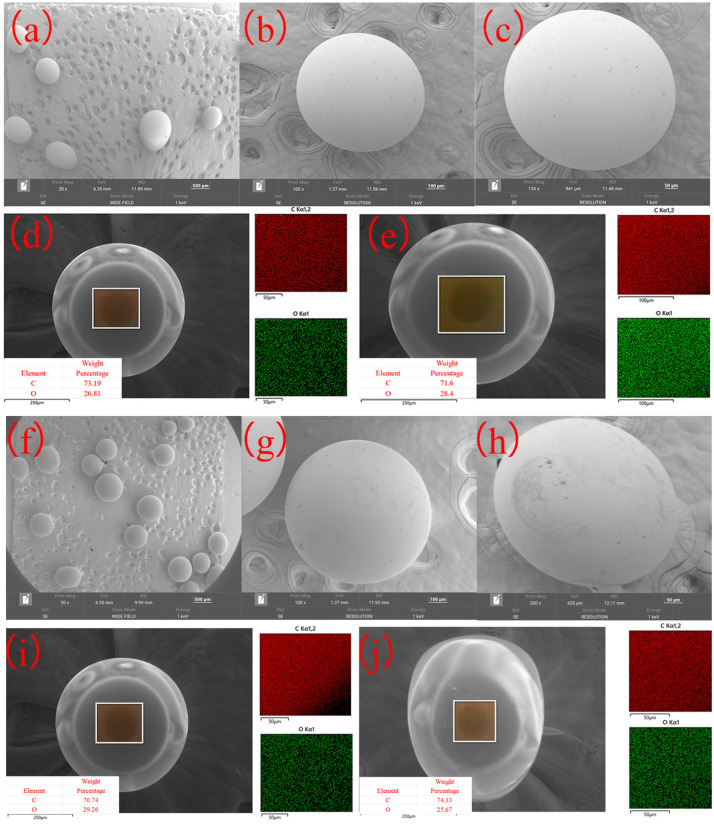
SEM micrographs (**a**–**c**,**f**–**h**) and corresponding EDS elemental maps (**d**,**e**,**i**,**j**) of the resin before (**a**–**e**) and after (**f**–**j**) adsorption of COD from semi-coking wastewater.

The surface morphology and elemental composition of the resin before and after adsorption of COD from semi-coking wastewater were examined using SEM and EDS, as shown in Figure 5. The pristine resin (Figure 5a–c) exhibits smooth, spherical microspheres with uniform morphology and dense surfaces, characteristic of well-polymerized carbon-based resins. No apparent cracks or surface irregularities are observed, suggesting mechanical stability and low porosity exposure at the outer shell. In contrast, after adsorption (Figure 5f–h), the microspheres show slightly roughened surfaces with the appearance of subtle textural features and shallow depressions. These morphological changes indicate surface interaction with adsorbed organics and partial clogging of accessible pores by macromolecular compounds from the wastewater.

The EDS mapping results (Figure 5d,e,i,j) confirm that C and O are the main surface elements both before and after adsorption, consistent with the resin’s carbonaceous composition and surface oxygen functionalities. Quantitatively, before adsorption, the carbon content was 71.6–73.19 wt%, while oxygen accounted for 26.81–28.4 wt%. After adsorption, the carbon proportion slightly varied to 74.33–70.74 wt%, accompanied by oxygen contents of 25.67–29.26 wt%. These minor fluctuations suggest the adsorption of oxygen-rich organic molecules and possible redox interaction between surface functional groups and wastewater constituents.

The EDS elemental mapping further shows homogeneous distributions of both C and O over the microsphere surface, confirming that the adsorption process occurs uniformly rather than being localized to specific surface sites. The slight increase in oxygen signal intensity after adsorption corresponds to the attachment of oxygenated organics—such as phenolic, carboxylic, and carbonyl compounds—identified by FTIR analysis.

Overall, the SEM–EDS characterization demonstrates that the resin maintains its spherical morphology after adsorption, with only minor surface roughening. The stable morphology combined with the uniform elemental distribution indicates good structural robustness, while the observed oxygen enrichment corroborates the hydrogen-bonding interactions involved in COD removal from semi-coking wastewater.

### 2.2. Adsorption Performance

#### 2.2.1. The Effect of Resin Amount

**Figure 6:**
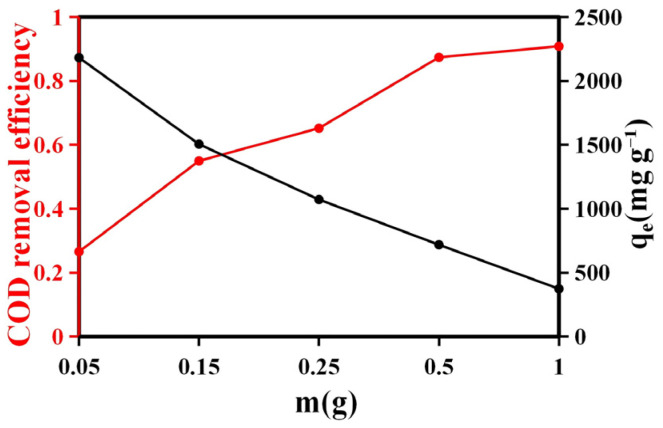
Effect of resin dosage on COD removal efficiency and adsorption capacity (*qₑ*). Experimental conditions: adsorption time of 1440 min, initial COD concentration of 82,170 mg L^−1^, and temperature of 50 °C.

The effect of resin dosage on the adsorption performance for COD removal from semi-coking wastewater was evaluated, and the results are shown in Figure 6 and Table S1. As the resin mass increased from 0.05 g to 1.0 g, the COD removal efficiency increased significantly from 27% to 91%, whereas the adsorption capacity decreased from 2182 mg g^−1^ to 373.2 mg g^−1^.

This opposite trend between removal efficiency and adsorption capacity reflects the balance between the number of available active sites and the concentration of adsorbate molecules. At lower resin dosages, the amount of adsorbent is insufficient to capture all organics in solution, leading to lower overall removal efficiency. However, the limited number of sites becomes highly saturated, resulting in a relatively high adsorption capacity per unit mass of resin. As the dosage increases, more adsorption sites become available, allowing more efficient capture of organic pollutants and a substantial increase in removal efficiency.

Nevertheless, the adsorption capacity per gram of resin decreases with increasing dosage. This phenomenon is primarily attributed to adsorption site underutilization and particle aggregation, which reduce the effective surface area and diffusion accessibility of active sites. Moreover, the overlapping of the boundary layers around individual resin particles at higher dosages leads to mass transfer limitations, further lowering the apparent adsorption capacity.

These results suggest that an optimal resin dosage exists at which COD removal efficiency and adsorption utilization are balanced, while also considering practical factors such as material consumption and operational cost. Based on this criterion, 0.5 g of resin was selected as the optimal dosage, achieving high removal efficiency (≈87%) with relatively effective surface utilization. Preliminary experiments were repeated multiple times to ensure the stability and reliability of the results. After confirming consistent trends, the subsequent series experiments were conducted under identical conditions.

The variation in adsorption performance with resin dosage demonstrates that COD removal from semi-coking wastewater is governed by the interplay between available adsorption sites, mass transfer resistance, and adsorbate–adsorbent interactions, consistent with typical heterogeneous adsorption behavior observed for porous carbonaceous materials.

#### 2.2.2. The Effect of Time and Temperature

**Figure 7:**
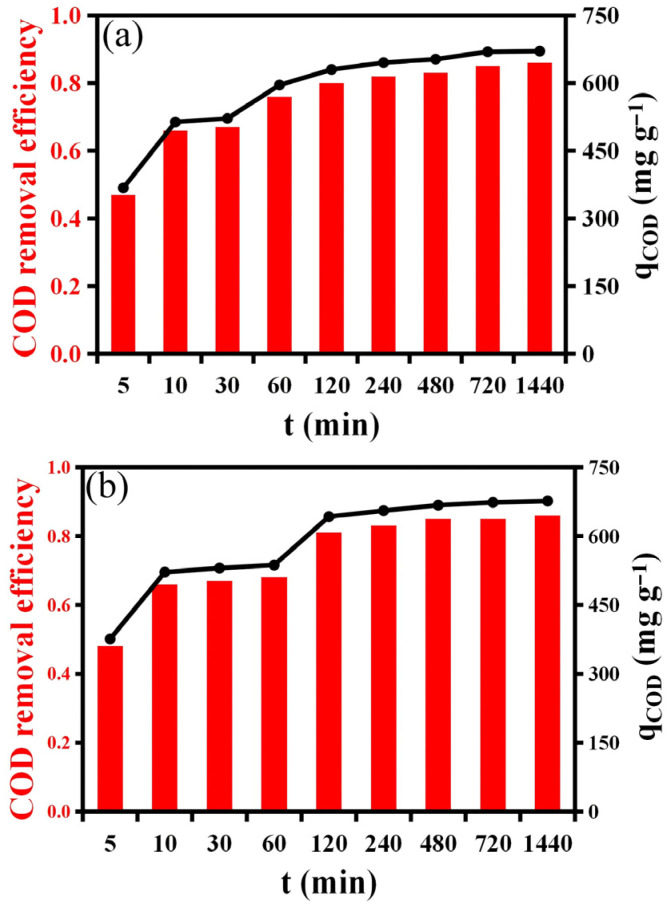

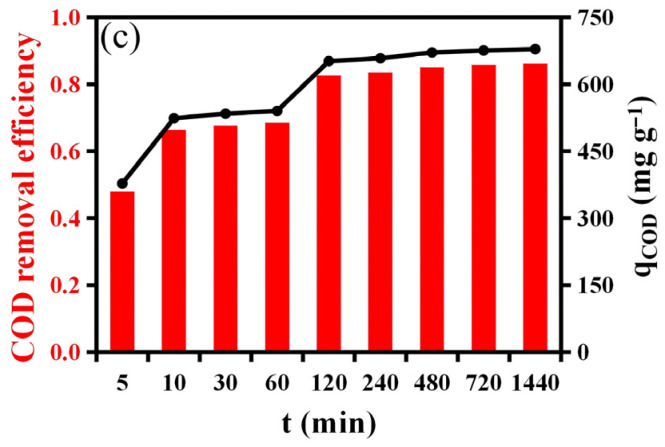
Adsorption kinetics curves showing the effect of contact time and temperature on COD removal efficiency and adsorption amount on 0.5 g resin at different temperatures: (**a**) 30°, (**b**) 40°, and (**c**) 50°. The datasets correspond to independent measurements at each temperature, with similar trends reflecting the weak temperature dependence of the adsorption process.

The influence of contact time and temperature on COD removal and adsorption capacity was investigated using 0.5 g of resin, and the results are summarized in Figure 7 and Table S2. As shown in Figure 7a–c, both COD removal efficiency and adsorption capacity increased rapidly during the initial adsorption stage (within the first 120 min), followed by a gradual approach toward equilibrium after approximately 240–480 min. At 30 °C, the removal efficiency increased from 47% to 86%, and the adsorption amount rose from 367.5 mg g^−1^ to 670.8 mg g^−1^ as the contact time extended from 5 min to 1440 min. A similar trend was observed at 40 °C and 50 °C, where equilibrium was reached slightly earlier (around 240 min), suggesting that higher temperatures promote faster diffusion and interaction between COD molecules and active sites on the resin surface.

The initial rapid adsorption can be attributed to the abundance of available active sites and the high concentration gradient between the bulk solution and the resin surface. As adsorption progresses, these sites become gradually occupied, leading to a decrease in adsorption rate until equilibrium is reached. The slower adsorption rate in the later stages can also be explained by intraparticle diffusion resistance and pore saturation, consistent with a diffusion-controlled process.

Temperature was found to have a positive effect on COD removal efficiency (Figure 7). When the adsorption temperature increased from 30 °C to 50 °C, both the removal efficiency and equilibrium adsorption capacity exhibited a slight but consistent enhancement. For example, at 1440 min, the adsorption capacity increased from 670.8 mg g^−1^ (30 °C) to 678.7 mg g^−1^ (50 °C). This improvement indicates that the adsorption process is endothermic in nature, likely due to enhanced molecular mobility and reduced solution viscosity at elevated temperatures, which facilitate the diffusion of COD species into the resin pores. The minimal changes in equilibrium capacity across temperatures indicate that the process is primarily governed by surface diffusion and pore filling, rather than by chemisorption involving strong chemical bond formation.

The adsorption of COD from semi-coking wastewater onto the resin is a time-dependent and mildly endothermic process, characterized by an initial rapid surface adsorption stage followed by a slower intraparticle diffusion phase. The results confirm that higher temperatures slightly enhance adsorption kinetics without significantly affecting the overall capacity, indicating a physisorption process governed by diffusion and non-covalent interactions.

#### 2.2.3. Adsorption Isotherms

**Figure 8:**
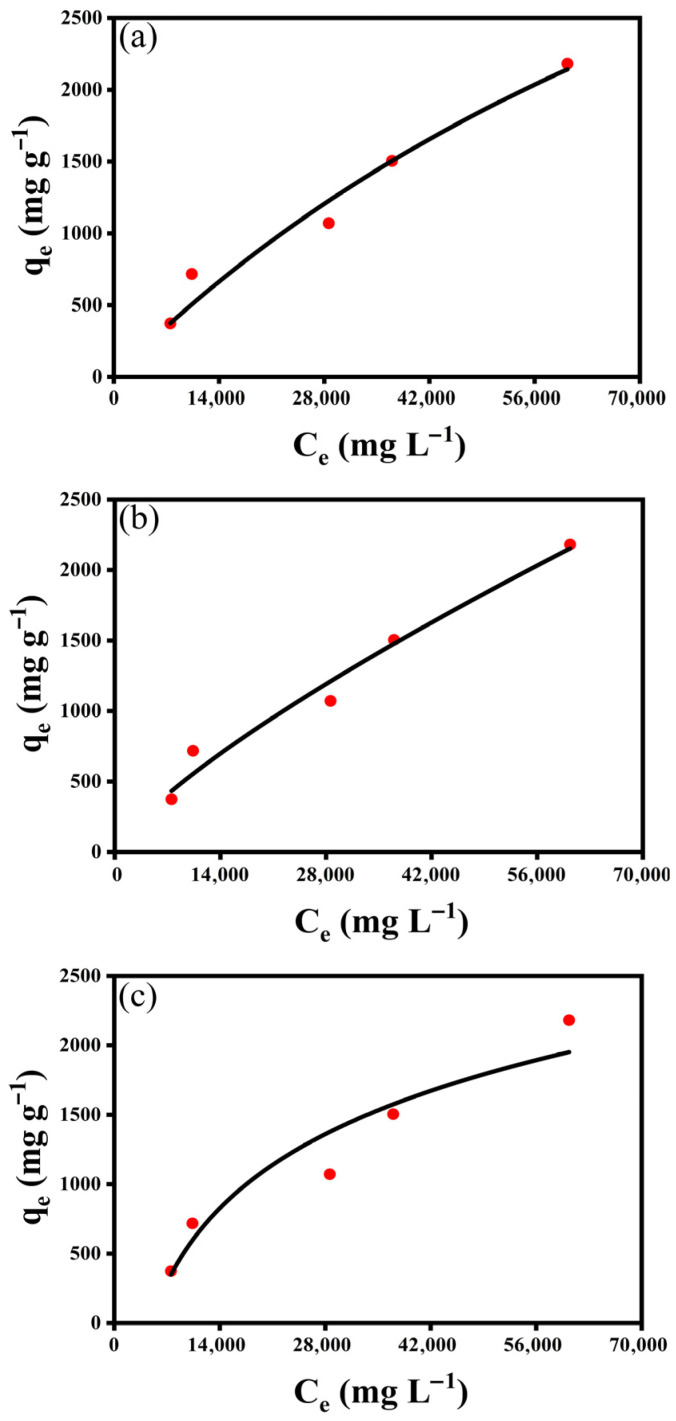
Adsorption isotherms of COD on the resin fitted using non-linear regression with (**a**) Langmuir model, (**b**) Freundlich model, and (**c**) Temkin model, respectively. Symbols represent experimental data, and solid lines represent the corresponding model fits.

The adsorption equilibrium data for COD removal by the resin were analyzed using the Langmuir, Freundlich, and Temkin isotherm models [41], as illustrated in Figure 8, with the corresponding parameters summarized in Table 3. In addition, the linearized fitting results are provided in Figure S1 and Table S4 for comparison. These models describe the distribution of adsorbate molecules between the liquid and solid phases at equilibrium and provide insight into the adsorption mechanism and surface characteristics of the adsorbent. In this study, both linear and non-linear regression methods were applied; however, non-linear fitting is considered more accurate, as it avoids the error distortion introduced by linearization.

The Langmuir isotherm model assumes monolayer adsorption on a homogeneous surface with a finite number of identical sites [27,42]. From the fitting results, q_max_ value was estimated to be 6534 mg g^−1^, with R^2^ of 0.9644, indicating a reasonable description of the adsorption behavior. The corresponding linear fitting results (Table S4) show lower consistency, suggesting that the assumptions of ideal monolayer adsorption are not fully satisfied.

The Freundlich isotherm model, an empirical equation describing adsorption on heterogeneous surfaces [43,44]. The non- linear fitting results yielded K_F_ = 0.44626 (mg g^−1^) (L mg^−1^)^−1/n^ and 1/n = 0.7705 with an R^2^ = 0.9742. The value of 1/n between 0 and 1 indicates favorable adsorption and reflects the heterogeneous nature of the resin surface. The linear fitting results show a similar trend but with slightly lower fitting accuracy.

The Temkin isotherm model accounts for the interactions between adsorbate and adsorbent and assumes a uniform distribution of binding energies across heterogeneous surface sites. The model of both linear and non-linear fitting produced k_1_ = 769.4 mg g^−1^ and k_2_ = 0.00021 L mg^−1^, with R^2^ = 0.9162, also indicating a reasonable fit to the experimental data. The positive value of k_2_ suggests that the adsorption process is endothermic and involves a gradual decrease in the heat of adsorption with increasing surface coverage, which is typical for physical adsorption involving π–π interactions and hydrogen bonding [45,46].

Overall, both linear and non-linear analyses indicate that the Freundlich model provides the best fit to the experimental data, followed by the Temkin model and the Langmuir model. However, the non-linear regression results are considered more reliable for interpreting the adsorption behavior. The results suggest that COD adsorption occurs on a heterogeneous surface with non-uniform energy distribution. Therefore, the adsorption behavior is dominated by heterogeneous surface interactions, consistent with the structural characteristics of the resin observed in BET and SEM analyses. The good correlation with the Freundlich and Temkin models further confirms that the adsorption process is mainly controlled by non-covalent interactions, such as π–π stacking and hydrogen bonding. It should be noted that semi-coking wastewater is a complex multicomponent system. Therefore, the isotherm models, which are primarily developed for single-component adsorption, are used here to describe overall adsorption trends rather than to provide a rigorous mechanistic interpretation of individual species.

#### 2.2.4. Kinetics Analysis

**Figure 9:**
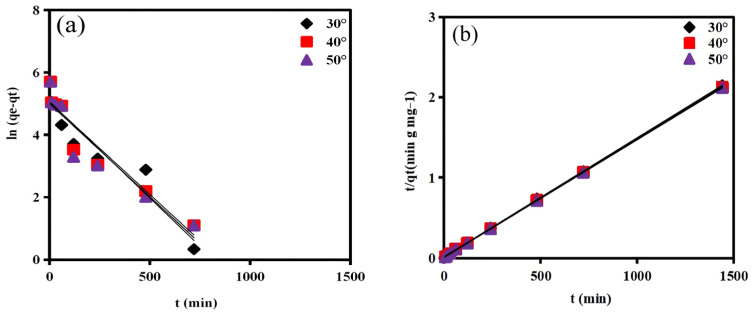

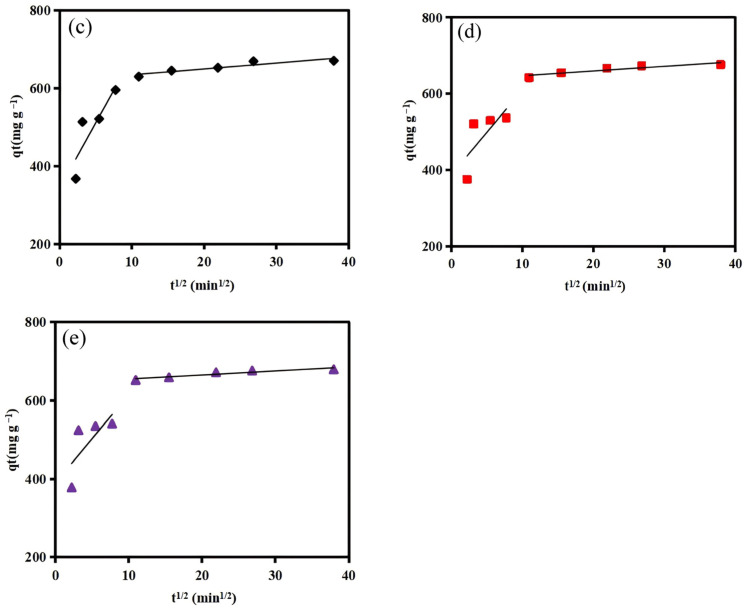
Adsorption kinetics modeling of COD uptake by the resin: (**a**) pseudo-first-order (PFO) kinetic model, (**b**) pseudo-second-order (PSO) kinetic model, and intra-particle kinetic model at (**c**) 30°, (**d**) 40°, and (**e**) 50°. Symbols represent experimental data, and lines represent model fittings.

To elucidate the adsorption mechanism and rate-controlling steps of COD removal by the resin, the kinetic data were analyzed using the pseudo-first-order (PFO), pseudo-second-order (PSO), and intraparticle diffusion (IPD) models [47,48]. The linear fitting results are presented in Figure 9 and summarized in Table 4, while the corresponding non-linear regression results are shown in Figure S2 and Table S5.

As observed in Figure 9 and Table 4, the PFO model, which assumes that the adsorption rate is proportional to the number of unoccupied sites, yielded moderate coefficient of determination (R^2^ = 0.8391–0.8583) and noticeable deviation between the calculated and experimental equilibrium adsorption capacities. This indicates that the PFO model does not accurately describe the COD adsorption process on the resin surface. Conversely, the PSO model, based on the assumption that the adsorption rate is governed by surface interaction or electron exchange, showed excellent agreement with the experimental data [49,50]. The coefficient of determination (R^2^ = 0.9999) and *q**_e_*** values (675.7–680.3 mg g^−1^) were highly consistent across all temperatures (30–50 °C), confirming that the adsorption kinetics of COD onto the resin follow the pseudo-second-order model. This implies that surface-controlled processes such as hydrogen bonding and π–π electron donor–acceptor interactions between aromatic compounds in the wastewater and the carbonaceous resin matrix are the primary mechanisms governing adsorption [35,44,48].

The non-linear regression results further support this trend. As shown in Figure S2 and Table S5, the PSO model consistently exhibits higher R^2^ values (0.8516–0.924) than the PFO model (0.7130–0.8018), along with reasonable agreement between calculated and experimental qₑ values. However, in this study, the linear fitting results demonstrate slightly better agreement with the experimental data, suggesting that the linear PSO model provides a more suitable description of the adsorption kinetics under the investigated conditions. Both linear and non-linear analyses indicate that the PSO model better describes the adsorption kinetics of COD on the resin.

To further examine diffusion behavior, the intraparticle diffusion model proposed by Weber and Morris was applied. The IPD plots exhibit two apparent linear regions, (Figure 9c–e), which are commonly interpreted as indicative of multiple stages in the adsorption process. The initial region is generally associated with rapid uptake at the external surface or boundary layer, while the later region may reflect diffusion of COD molecules into the internal pore structure of the resin [48,51]. However, it should be noted that the identification of such stages is based on visual interpretation and should be considered qualitative [52]. In the initial stage, the IPD rate constants (k_p1_) were 33.51, 22.25, and 22.57 mg g^−1^ min^−1^/^2^ at 30, 40, and 50 °C, respectively, with relatively high intercepts (C_1_ = 343.6–388.9 mg g^−1^) and moderate R^2^ values (0.7513–0.8580). The second stage showed smaller diffusion constants (k_p2_ = 1.512, 1.228, and 1.035 mg g^−1^ min^−1^/^2^) and higher intercepts (C_2_ = 619.5–643.7 mg g^−1^). These trends are consistent with a transition from faster initial uptake to slower mass transfer within the pore structure, although the exact mechanistic interpretation remains uncertain. The nonzero intercepts and the fact that the IPD plots do not pass through the origin suggest that intraparticle diffusion is not the only rate-controlling step. Therefore, the IPD model is used here primarily to provide qualitative insight into diffusion behavior, rather than to establish definitive rate-limiting mechanisms.

Overall, the kinetic analysis indicates that COD adsorption on the resin is well described by the pseudo-second-order model, suggesting that surface interactions such as hydrogen bonding and π–π interactions play an important role in the adsorption process. The intraparticle diffusion analysis provides qualitative evidence that diffusion within the pore structure may influence the later stages of uptake; however, it should not be considered as a definitive rate-controlling mechanism. These findings are consistent with the FTIR, XPS, and BET results, which collectively suggest that adsorption involves both pore diffusion and specific surface interactions between the resin and organic constituents of semi-coking wastewater.

#### 2.2.5. Thermodynamic Analysis

**Figure 10:**
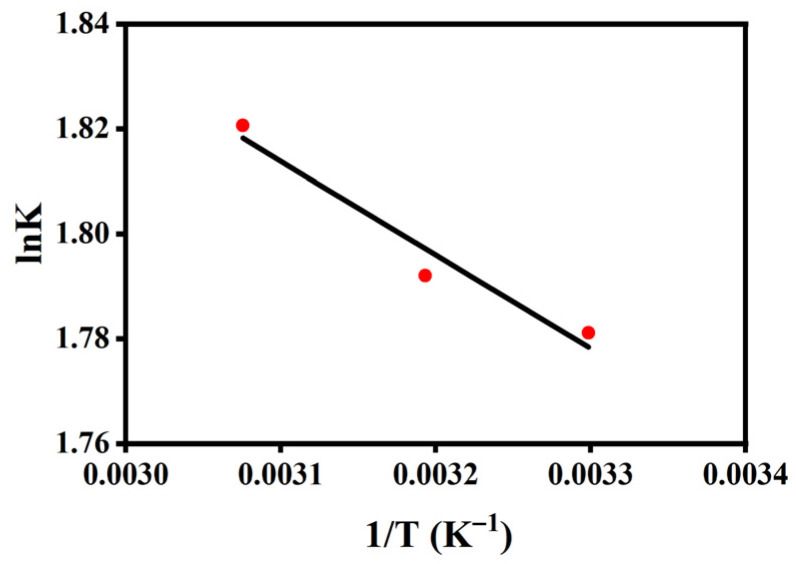
van’t Hoff plot for the adsorption of COD on resin.

To further elucidate the nature of COD adsorption on the resin, thermodynamic parameters including the Gibbs free energy change (ΔG°), enthalpy change (ΔH°), and entropy change (ΔS°) were evaluated from the temperature-dependent adsorption data (Figure 10 and Table 5). The thermodynamic calculations were performed using a dimensionless equilibrium constant, as described in the Section 3, to ensure appropriate interpretation. As shown in Table 5, the values of ΔG° are −4.489, −4.666, and −4.922 kJ mol^−1^ at 303.15 K, 313.15 K, and 323.15 K, respectively. The negative ΔG° values indicate that the adsorption process is thermodynamically favorable under the studied conditions. The slightly more negative ΔG° values at higher temperatures suggest a modest enhancement in adsorption affinity with increasing temperature.

The calculated ΔH° value of 1.483 kJ mol^−1^ is positive; however, its very small magnitude should be interpreted with caution. This near-zero enthalpy may reflect the limited temperature range and fitting constraints rather than the exact thermodynamic nature of the adsorption process. Nevertheless, the relatively low enthalpy value (<40 kJ mol^−1^) suggests that the process is likely dominated by weak physical interactions, such as electrostatic interactions, van der Waals forces, and hydrogen bonding, rather than strong chemisorption [44,53]. A more rigorous thermodynamic analysis would require isostere-based evaluation at constant surface coverage, as suggested in the literature [54].

The positive ΔS° value (19.68 J mol^−1^ K^−1^) suggests an apparent increase in interfacial disorder during adsorption. In complex systems such as semi-coking wastewater, this behavior may be associated with solvent displacement, structural rearrangement at the interface, and the heterogeneous nature of the adsorbed species.

Overall, the thermodynamic analysis indicates that the adsorption process is favorable and primarily governed by physical interactions. However, due to the multicomponent nature of the wastewater and the limitations of the thermodynamic approach, these parameters should be regarded as approximate and interpreted in terms of general trends rather than definitive mechanistic conclusions. These findings remain consistent with the kinetic and spectroscopic results, which suggest that adsorption involves a combination of surface interactions and pore diffusion processes.

### 2.3. Mechanism and Future Directions

The integrated results from FTIR, XPS, BET, XRD, and SEM–EDS analyses collectively demonstrate that the adsorption of COD from semi-coking wastewater onto the porous carbon-based resin proceeds via a synergistic, primarily physisorptive mechanism involving multiple concurrent steps. Initially, COD-bearing organic molecules are transported from the bulk solution to the resin surface through film diffusion. This rapid process is supported by the kinetic analysis, in which the IPD model shows a steep initial linear region with larger *k*_*p*_ values (33.51, 22.25, and 22.57 mg g^−1^ min^−½^ at 30–50 °C), indicating efficient external mass transfer and abundant available adsorption sites at the early stage.

Once at the surface, adsorption proceeds through non-covalent interactions between functional groups on the resin and oxygenated aromatic compounds in the wastewater. FTIR spectra reveal that the broad –OH stretching band shifts from 3442 to 3435 cm^−1^ after adsorption, suggesting enhanced hydrogen bonding between the resin’s polar sites and phenolic or carboxylic groups from the wastewater. The growth of aromatic (3050 cm^−1^) and aliphatic (2924 and 2860 cm^−1^) C–H stretching bands, along with new carbonyl peaks at 1730 and 1677 cm^−1^, confirms the adsorption of aromatic and oxygenated organics. XPS analysis further corroborates the increase in surface C–O and C=O functionalities, demonstrating the involvement of hydrogen bonding, π–π interactions, and dipole–dipole interactions at the resin interface.

As adsorption continues, adsorption occurs on a heterogeneous surface. The Freundlich isotherm provided the best fit, indicating heterogeneous binding sites. The BET and t-plot analyses also reveal a substantial decrease in surface area (1306.22 → 825.99 m^2^ g^−1^) and pore volume (0.562 → 0.421 cm^3^ g^−1^) after adsorption, confirming that pore filling and internal diffusion play key roles in the overall process. In the second stage of the IPD plots, smaller *kp* values (1.0–1.5 mg g^−1^ min^−½^) and higher intercepts (C ≈ 620–644 mg g^−1^) further indicate slower intraparticle diffusion and the gradual occupation of inner pores as equilibrium is approached; however, this interpretation remains qualitative.

The XRD results show two broad diffraction peaks at 15.36° and 41.29°, typical of amorphous turbostratic carbon. After adsorption, these peaks weaken and slightly shift, indicating increased structural disorder due to organic deposition without altering the carbon backbone. SEM observations reveal that the spherical morphology of the resin remains intact, although the surface becomes slightly roughened after adsorption. EDS mapping shows uniform C and O distributions, with minor changes in elemental composition (C = 71.6–73.2 wt% before, 70.7–74.3 wt% after). Thermodynamic analysis further supports this interpretation. The negative ΔG° values (−4.66 to −4.92 kJ mol^−1^) indicate that the adsorption process is thermodynamically favorable. The calculated ΔH° value is small and positive, but should be interpreted with caution due to the limited temperature range and the multicomponent nature of the wastewater. The positive ΔS° suggests an apparent increase in interfacial disorder, which may be associated with desolvation effects and the heterogeneous nature of the adsorbed species.

Overall, the adsorption of COD from semi-coking wastewater onto the resin can be described as a multi-step process involving (i) external mass transfer, (ii) surface interactions dominated by π–π interactions and hydrogen bonding, and (iii) pore diffusion and filling within the hierarchical structure. The process is primarily governed by physical interactions rather than chemical bond formation.

It should be noted that semi-coking wastewater is a complex multicomponent system. Therefore, the proposed mechanism represents a generalized interpretation based on combined experimental evidence, and the applied models describe overall adsorption trends rather than specific interactions of individual compounds.

Looking ahead, several research directions can further improve understanding and performance. First, regeneration and stability studies are necessary to evaluate reusability across multiple adsorption–desorption cycles, tracking surface chemistry and structure by FTIR, XPS, and BET analyses. Second, the effects of solution chemistry (e.g., pH, ionic strength, and competing species) should be systematically investigated to understand selectivity and real wastewater performance. Third, dynamic column experiments are recommended to complement batch studies and support process scale-up. In addition, more rigorous thermodynamic analysis based on adsorption isosteres is needed to obtain reliable enthalpy values. Advanced in situ characterization techniques (e.g., Raman spectroscopy and solid-state NMR) could further elucidate adsorption interactions at the molecular level.

From a materials perspective, future work may focus on tuning surface functionality (e.g., controlled oxidation or heteroatom doping) and optimizing pore structure to enhance accessibility and selectivity for complex organic mixtures. Integrating this resin with advanced oxidation or biological post-treatment may also improve overall treatment efficiency. Finally, life-cycle and techno-economic assessments are recommended to evaluate the feasibility and sustainability of this adsorption technology.

## 3. Materials and Methods

### 3.1. Materials and Reagents

The commercial porous carbon-based resin used as the adsorbent was kindly supplied by Sunresin New Materials Co., Ltd., (Xi’an, China). The physicochemical properties of the resin have been described in our previous publication. The semi-coking wastewater sample was obtained from a coal chemical industry located in Yulin, Shaanxi Province, China. The wastewater exhibited a dark brown color, with an initial COD concentration of approximately 50,000 mg L^−1^, while its ionic strength was not determined separately and is assumed to reflect the intrinsic composition of the semi-coking wastewater. All glassware was thoroughly cleaned and pre-rinsed with the COD-containing solution to minimize adsorption losses on container walls.

### 3.2. Batch Adsorption Experiments

Batch adsorption experiments were performed to evaluate the removal efficiency and adsorption behavior of COD from semi-coking wastewater. The experimental design focused on elucidating the adsorption mechanism through analysis of isotherms, kinetics, and thermodynamics. The initial pH of the wastewater was approximately 9.0, and the initial COD concentration was around 50,000 mg L^−1^.

#### 3.2.1. Effect of Resin Dosage

To investigate the influence of resin dosage on COD removal efficiency, five 15 mL centrifuge tubes were prepared, each containing a different amount of resin (0.05, 0.15, 0.25, 0.50, and 1.00 g). Subsequently, 5 mL of semi-coking wastewater was added to each tube. The tubes were sealed with parafilm to prevent evaporation and placed in a thermostatic shaker set at 50 °C and 150 rpm for 1440 min to achieve equilibrium adsorption. After the reaction period, the mixtures were filtered to remove resin particles, and the supernatants were collected for COD analysis using a multiparameter water analyzer (Model YKM-N3, Shanghai, China).

#### 3.2.2. Effect of Contact Time and Temperature

To examine the effects of contact time and temperature on COD adsorption, kinetic and thermodynamic experiments were conducted using 0.5 g of resin at three temperatures: 30, 40, and 50 °C. In each trial, 5 mL of semi-coking wastewater was added to the adsorption tubes containing the resin. The tubes were agitated in a thermostatic shaker (THZ-C, Changzhou, China) at 150 rpm, and samples were withdrawn at specified time intervals (5, 10, 30, 60, 120, 240, 480, 720, and 1440 min). After shaking, each suspension was immediately filtered, and the supernatant was analyzed for residual COD concentration using the YKM-N3 multiparameter water analyzer.

The equilibrium constant (*K*), COD removal efficiency (ω), adsorption capacity (*q**_e_***, mg g^−1^), and adsorption amount (*q**_COD_*** = *q**_t_***, mg g^−1^) were calculated using the following equations:

(1) $$K\;=\;\frac{q_{e}}{C_{e}}$$

(2) $$\omega \;=\frac{\left(C_{0\;}-\;C_{t}\right)}{C_{0}}$$

(3) $$qe=\frac{{(C}_{0}\;-\;C_{e})\;\times \;V}{m}$$

(4) $$q_{\mathrm{COD}}=q_{t}=\frac{{(C}_{0}\;-\;C_{t})\;\times \;V}{m}$$

where *C***_0_** is the initial COD concentration (mg L^−1^), *C****ₜ*** is COD concentration at time t (min), *C****ₑ*** is the equilibrium COD concentration (mg L^−1^), *V* is the solution volume (5 mL), *m* is the mass of the resin (g). These formulas were consistently applied in all adsorption experiments to calculate the adsorption kinetics, isotherm parameters, and thermodynamic properties for the resin in removing COD from semi-coking wastewater.

The methodologies used for structural characterization (FTIR, XPS, BET, XRD, and SEM–EDS) and the procedures for adsorption isotherm, kinetic, and thermodynamic analyses followed previously reported literature methods with appropriate modifications for this study [27].

## 4. Conclusions

This study systematically investigated the adsorption performance and mechanism of a porous carbon-based resin for the removal of COD from semi-coking wastewater. Structural characterization confirmed that the resin possesses a highly porous amorphous carbon framework with abundant oxygen-containing functional groups and a hierarchical micro–mesoporous architecture, providing favorable conditions for the adsorption of complex organic pollutants.

Spectroscopic analyses (FTIR and XPS) revealed that surface hydroxyl, carbonyl, and ether functionalities participate in the adsorption process through hydrogen bonding and π–π interactions with aromatic and oxygenated compounds present in the wastewater. Textural characterization showed significant decreases in surface area and pore volume after adsorption, indicating that pore filling and internal diffusion play important roles in COD capture. XRD and SEM–EDS results further demonstrated that the resin maintains structural integrity after adsorption while experiencing slight surface roughening and enrichment of oxygen-containing species due to organic deposition.

Adsorption studies showed that the resin exhibits excellent COD removal performance, achieving a maximum removal efficiency of approximately 91%. The adsorption kinetics follow the pseudo-second-order model (R^2^ ≈ 0.9999), suggesting that surface interactions dominate the adsorption rate. Equilibrium data are best described by the Freundlich isotherm model (R^2^ = 0.9742), indicating heterogeneous multilayer adsorption. Thermodynamic analysis confirmed that the adsorption process is spontaneous (ΔG° < 0) and accompanied by increased interfacial disorder (ΔS° > 0), while the positive but near-zero enthalpy value (ΔH° > 0) should be interpreted with caution due to possible limitations in the fitting and temperature range; overall, the process is consistent with a mechanism primarily governed by physical adsorption.

COD removal by the porous carbon-based resin occurs through a synergistic mechanism involving pore filling, π–π interactions, and hydrogen bonding within the hierarchical carbon framework. The high adsorption efficiency and structural stability demonstrate the strong potential of this resin as an effective adsorbent for the treatment of high-strength industrial wastewater such as semi-coking effluents. Due to the multicomponent nature of semi-coking wastewater, the applied models describe overall adsorption behavior rather than specific interactions of individual compounds. These findings provide valuable mechanistic insight and contribute to the development of efficient adsorption materials for complex wastewater remediation.

## Figures and Tables

**Table 1:** Elemental composition and surface chemical states of the resin before and after adsorption of semi-coking wastewater, as determined by XPS.

	Binding Energy	Before	Binding Energy	After
	(eV)	(%)	(eV)	(%)
Surface concentration				
C1s		92.29		90.63
O1s		7.710		7.760
N1s				1.610
C Surface concentration [34,35]				
C=C/C–C	284.8	81.92	284.8	69.50
C–O	286.1	14.80	286.1	25.31
C=O	287.7	3.28	287.7	5.190
π–π*	291.4	/	291.1	/
O Surface concentration [36]				
C=O	532.0	37.39	532.3	38.71
C–O	533.3	51.48	533.6	48.94
H_2_O	534.9	11.13	535.1	12.35

**Table 2:** Porous structural properties of the resin before and after adsorption of semi-coking wastewater.

	BET Surface Area	Langmuir Surface Area	BJH Adsorption Surface Area	t-Plot Micropore Area	BJH Adsorption Volume	t-Plot Micropore Volume
	m^2^ g^−1^	m^2^ g^−1^	m^2^ g^−1^	m^2^ g^−1^	cm^3^ g^−1^	cm^3^ g^−1^
before	1306	2340	324.2	617.4	0.562	0.2505
after	826	1594	213	371.3	0.421	0.1509

**Table 3:** Parameters of Langmuir, Freundlich, and Temkin isotherm models obtained by non-linear regression for COD adsorption on the resin.

Langmuir Parameters			Freundlich Parameters			Temkin Parameters		
q_max_ (mg g^−1^)	b (L mg^−1^)	R^2^	K_F_ (mg g^−1^) (L mg^−1^)^1/n^	1/n	R^2^	k_1_ (mg g^−1^)	k_2_	R^2^
6534	0.00000808	0.9644	0.44626	0.7705	0.9742	769.4	0.00021	0.9162

**Table 4:** The fitted parameters of kinetic models for COD adsorption on 0.5 g resin.

		Unit	30 °C		40 °C		50 °C	
PFO	k_1_	min^−1^	0.00618		0.00591		0.00597	
	q_e_	mg g^−1^	158.5		155.2		148.6	
	R^2^		0.8405		0.8583		0.8391	
PSO	k_2_	g mg^−1^ min^−1^	0.000193		0.000186		0.000196	
	q_e_	mg g^−1^	675.7		680.3		680.3	
	R^2^		0.9999		0.9999		0.9999	
IPD	k_p_	mg g^−1^ min^−1/2^	33.51	1.512	22.25	1.228	22.57	1.035
	C	mg g^−1^	343.6	619.5	387.7	635.3	388.9	643.7
	R^2^		0.7513	0.858	0.5082	0.8413	0.5132	0.8649

**Table 5:** Thermodynamic fitting parameters for COD removal by 0.5 g resin.

T	ΔG	ΔH	ΔS	R^2^
(K)	(kJ mol^−1^)	(kJ mol^−1^)	(J mol^−1^ K^−1^)	
303.15	−4.489	1.483	19.68	0.9526
313.15	−4.666			
323.15	−4.922			

## Data Availability

The data of this study are available from the corresponding author upon reasonable request.

## Supplementary Materials

The following supporting information can be downloaded at: https://www.mdpi.com/article/10.3390/molecules31081319/s1, Figure S1: Linearized adsorption isotherm plots of COD on the resin based on (a) Langmuir, (b) Freundlich, and (c) Temkin models; Figure S2: Non-linear regression fitting of adsorption kinetics for COD on the resin using (a) pseudo-first-order (PFO) and (b) pseudo-second-order (PSO) models at different temperatures; Table S1: Effect of resin dosage on the removal efficiency and adsorption amount for COD in semi-coking wastewater; Table S2: Effect of contact time and temperature on the COD removal efficiency and adsorption amount of the resin; Table S3: Experimental dataset used for intraparticle diffusion model analysis of COD adsorption on the resin at different temperatures; Table S4: Isotherm parameters for COD adsorption on the resin obtained from linearized fitting of the Langmuir, Freundlich, and Temkin models; Table S5: Kinetic parameters for COD adsorption on the resin obtained from non-linear regression fitting of pseudo-first-order (PFO) and pseudo-second-order (PSO) models at different temperatures.
