# Supplementary material for: Spectroscopic and Thermodynamic Elucidation of COD Adsorption Mechanisms on a Porous Carbon-Based Resin
Source: Molecules. 2026 Apr 17;31(8):1319. doi: 10.3390/molecules31081319 (PMC13118468; doi:10.3390/molecules31081319)
Supplement: Supplementary file 1 [file molecules-31-01319-s001.zip › molecules-4218311-supplementary.pdf]

Table S1. Effect of resin dosage on the removal efficiency and adsorption amount for COD in semi-coking wastewater.

m	COD Removal Efficiency	q_{COD}
(g)	(%)	(mg g-1)
0.05	27	2182
0.15	55	1505
0.25	65	1072
0.5	87	717.9
1	91	373.2

Table S2. Effect of contact time and temperature on the COD removal efficiency and adsorption amount of the resin.

T	t	COD Removal Efficiency	q_{COD}
(°C)	(min)		(mg g^{-1})
30	5	0.47	367.5
	10	0.66	513.7
	30	0.67	521.5
	60	0.76	595.7
	120	0.8	630
	240	0.82	645.3
	480	0.83	652.9
	720	0.85	669.4
	1440	0.86	670.8
40	5	0.48	376
	10	0.66	521.5
	30	0.67	530.5
	60	0.68	537
	120	0.81	642.5
	240	0.83	655.5
	480	0.85	667.5
	720	0.85	673.5
	1440	0.86	676.5
50	5	0.4789	377.7
	10	0.664	523.7
T	t	COD Removal	q_{COD}

T	t	COD Removal Efficiency	q_{COD}
(℃)	(min)		(mg g^{-1})
		**Efficiency**	
(℃)	(min)		(mg g^{-1})
	30	0.6774	534.2
	60	0.6851	540.3
	120	0.8263	651.6
	240	0.8346	658.2
	480	0.8511	671.2
	720	0.8568	675.7
	1440	0.8606	678.7

Table S3. Experimental dataset used for intraparticle diffusion model analysis of COD adsorption on the resin at different temperatures.

t	$t^{1/2}$	q_t(mg g-1)		
		30℃	40℃	50℃
5	2.236	367.49	375.98	377.7
10	3.162	513.7	521.49	523.7
30	5.477	521.5	530.45	534.2
60	7.746	595.7	537.0	540.3
120	10.95	630.0	642.45	651.6
240	15.49	645.3	655.45	658.2
480	21.91	652.9	667.45	671.2
720	26.83	669.4	673.45	675.7
1440	37.94	670.8	676.45	678.7

Table S4. Isotherm parameters for COD adsorption on the resin obtained from linearized fitting of the Langmuir, Freundlich, and Temkin models.

Langmuir parameters			Freundlich parameters			Tempkin parameters		
q_{max} (mg g^{-1})	b (L mg^{-1})	R^2	K_F(mg g^{-1}) (L mg^{-1})$^{1/n}$	1/n	R^2	k_1 (mg g^{-1})	k_2	R^2
4920	0.0000125	0.6472	0.5159	0.7564	0.9445	769.4	0.00021	0.9162

Table S5. Kinetic parameters for COD adsorption on the resin obtained from non-linear

regression fitting of pseudo-first-order (PFO) and pseudo-second-order (PSO) models at different temperatures.

		Unit	30℃	40℃	50℃
PFO	k_1	min^{-1}	0.1698	0.1779	0.1772
	q_e	$mg\ g^{-1}$	627.9	627.2	631.1
	R^2		0.8018	0.7130	0.7174
PSO	k_2	$g\ mg^{-1}\ min^{-1}$	0.0004	0.0004	0.0004
	q_e	$mg\ g^{-1}$	653.0	653.1	657.1
	R^2		0.924	0.8516	0.8545

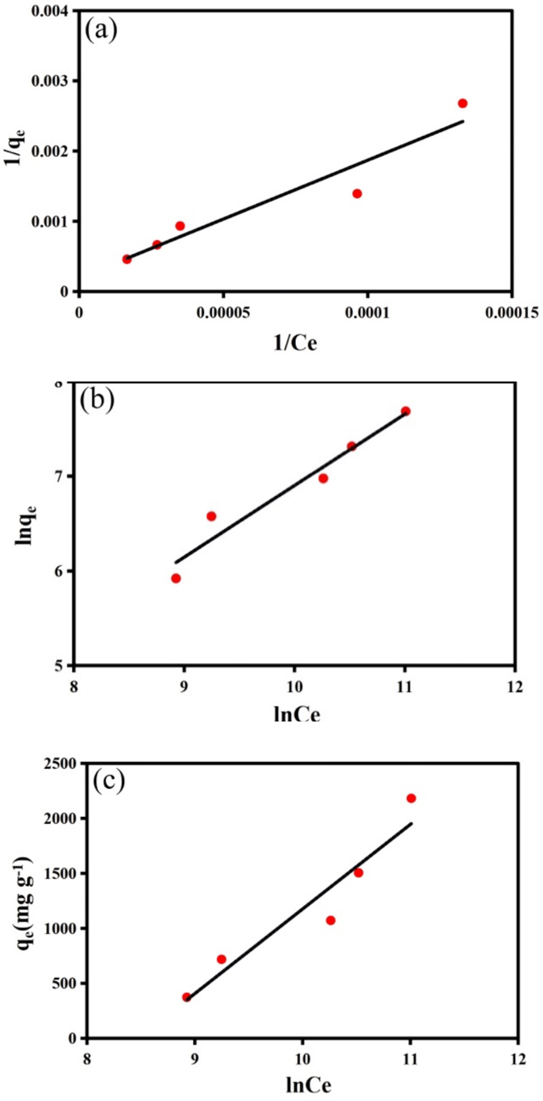

Figure S1. Linearized adsorption isotherm plots of COD on the resin based on

(a) Langmuir, (b) Freundlich, and (c) Temkin models.

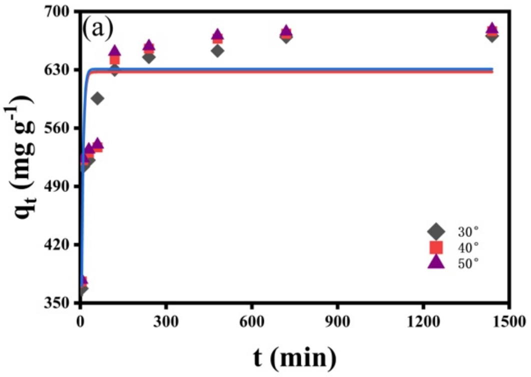

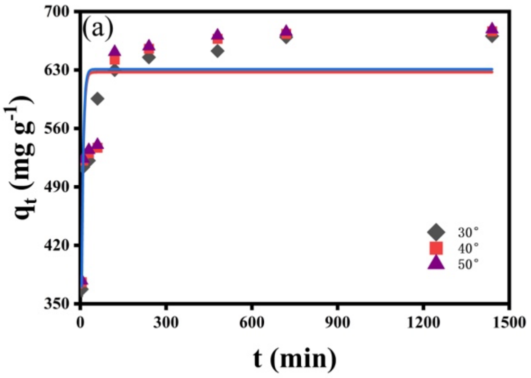

Figure S2. Non-linear regression fitting of adsorption kinetics for COD on the resin using (a) pseudo-first-order (PFO) and (b) pseudo-second-order (PSO) models at different temperatures.
